# Fever without a source in children: international comparison of guidelines

**DOI:** 10.1007/s12519-022-00611-8

**Published:** 2022-10-26

**Authors:** Sanne Graaf, Maya Wietske Keuning, Dasja Pajkrt, Frans Berend Plötz

**Affiliations:** 1 Department of Pediatrics, Tergooi Hospital, Rijksstraatweg 1, 1261 AN Blaricum, The Netherlands; 2 Department of Pediatrics, Amsterdam University Medical Centers, Location Academic Medical Center, University of Amsterdam, Amsterdam, The Netherlands; 3 Department of Pediatric Infectious Diseases, Amsterdam University Medical Centers, Location Academic Medical Center, University of Amsterdam, Amsterdam, The Netherlands

**Keywords:** Children, Fever, Guideline

## Abstract

**Background:**

Fever without a source (FWS) in children poses a diagnostic challenge. To distinguish a self-limiting infection from a serious infection, multiple guidelines have been developed to aid physicians in the management of FWS. Currently, there is no comparison of existing FWS guidelines.

**Methods:**

This comparative review describes consistencies and differences in guideline definitions and diagnostic and therapeutic recommendations. A literature search was performed to include secondary care FWS guidelines of high-income countries, composed by national or regional pediatric or emergency care associations, available in English or Dutch.

**Results:**

Ten guidelines of five high-income countries were included, with varying age ranges of children with FWS. In children younger than one month with FWS, the majority of the guidelines recommended laboratory testing, blood and urine culturing and antibiotic treatment irrespective of the clinical condition of the patient. Recommendations for blood culture and antibiotic treatment varied for children aged 1–3 months. In children aged above three months, urine culture recommendations were inconsistent, while all guidelines consistently recommended cerebral spinal fluid testing and antibiotic treatment exclusively for children with a high risk of serious infection.

**Conclusions:**

We found these guidelines broadly consistent, especially for children with FWS younger than one month. Guideline variation was seen most in the targeted age ranges and in recommendations for children aged 1–3 months and above three months of age. The findings of the current study can assist in harmonizing guideline development and future research for the management of children with FWS.

**Supplementary Information:**

The online version contains supplementary material available at 10.1007/s12519-022-00611-8.

## Introduction

Fever is one of the most common pediatric presentations at the emergency department [1–3]. While most children recover spontaneously without treatment from a self-limiting infection, serious infections can be harmful with long-term sequelae and mortality as potential consequences. The incidence of serious bacterial infections in children up to three months is estimated at 8%, in neonates even higher at 9% to 13% [4–6]. Fever without a source (FWS), defined as acute fever since less than seven days without a clear focus of infection after a complete examination, is challenging for physicians as children often present with nonspecific symptoms and the initial clinical presentation can vary widely [7]. This can result in a delay in admission and treatment for serious infections. Multiple laboratory tests in search for a cause of FWS and empirical antibiotics are therefore applied frequently [8].

To overcome this diagnostic challenge, several countries have developed guidelines to guide physicians in the management and evaluation of FWS [8]. They aim to detect those children at risk for serious infections requiring immediate treatment while avoiding overuse of unnecessary investigations and therapies. Despite the availability of guidelines for both children with a presumed low or high risk of serious infection based on the clinical condition, the variability in definition and management of FWS is significant [9–13]. For example, hospital admission and cerebral spinal fluid (CSF) analysis rates vary across hospitals from 40% to 90% of children presenting with FWS [14]. This variation in practice could be explained by several factors, including inconsistency in diagnostic and treatment recommendations between FWS guidelines or low adherence to these guidelines [15].

The aim of this study was therefore to compare the definitions and the diagnostic and therapeutic recommendations published in national and regional FWS guidelines of high-income countries. Identifying differences between national guidelines with similar health care settings could improve harmonization of practice recommendations and inform future guideline development. With this approach, we aim to increase international consensus in high-income countries in definition and management of FWS.

## Methods

### Study design

A literature search was performed to identify national and regional FWS guidelines in children in high-income countries. Since there is no universal consensus on health care system quality classification, we defined several criteria to include high-income countries with comparable health care systems: (1) classified as high-income economy level by the 2020 World Bank classifications; (2) rated in the top 50 countries with highest life expectancy; and (3) an antibiotic drug resistance index of 50 or less [16–18].

The search was conducted on April 21th 2021, combining the search terms “guideline”, “child” and “fever” in the databases of PubMed, Web of Science and EBSCOhost, including variations of these terms (Supplementary Table 1). Only the most updated versions of guidelines were included. References of included articles were screened for eligibility using the forward and backward snowball method. Articles were included if a national or regional guideline described recommendations or considerations for children with FWS aged 0–18 years, including both children with a presumed low or high risk of serious infection based on the clinical condition. The recommendations had to describe FWS management, aiming to timely diagnose and treat serious infections. Guidelines had to be composed by national or regional pediatric or emergency care associations, health institutes, health networks or statewide health services and based on peer reviewed evidence or group consensus. The exclusion criteria were specified as follows: (1) local guidelines of hospitals; (2) primary care guidelines; (3) guidelines describing fever with a focus, fever of unknown origin (fever > seven days), early onset neonatal sepsis (within 72 hours after birth), a hospital-acquired fever or post-operative fever; (4) inclusion of both adults and children; (5) guideline not available in English or Dutch.

### Outcome parameters

Primary outcome was to describe consistencies and differences in (1) guideline definitions; (2) diagnostic recommendations; and (3) therapeutic recommendations of included guidelines. Guideline definitions were described for age of population, fever, FWS, potential serious infections and objectives. Diagnostic recommendations were compared for laboratory testing of white blood cell count (WBC), C-reactive protein (CRP), performing blood culture, urine culture, CSF analysis, and polymerase chain reaction assay (PCR) for viral pathogens such as influenza virus, respiratory syncytial virus, enterovirus and parechovirus. Therapeutic recommendations were compared for empirical intravenous antibiotic treatment and empirical intravenous acyclovir treatment. Empirical antibiotic treatment is defined as antibiotics that are administered prior to the identification of the causing pathogen.

We divided diagnostic and therapeutic recommendations into those advised to perform or those advised to consider. To further specify the target population, categories were established a priori to distinguish recommendations based on age irrespective of the clinical condition or based on age combined with clinical criteria. Categories were defined as follows: recommendations (1) advised for all children irrespective of the clinical condition; (2) advised for children with a high risk of infection; (3) advised for children with an intermediate risk of infection; or (4) advised for children with a low risk of infection. Clinical criteria for low, intermediate or high risk of serious infection were described per guideline.

## Results

A total of ten guidelines were included, four from Australia, three from the USA and one from the Netherlands, the UK and Canada, respectively (Table 1). The flowchart of the search is presented in Supplementary Fig. 1. The publication year of these guidelines ranged from 1993 to 2021. Five of ten guidelines reported an established method to grade the quality of evidence supporting their recommendations [19, 20, 22–24]. Six guidelines were composed by national associations or health institutes [19–23, 28].

**Table 1:** Guideline characteristics

Country	Guideline	Year of publication	Organization	Approach to rate quality of evidence
The Netherlands	Fever in children [19]	2013	Dutch Association of Pediatrics	AGREE II
United Kingdom	Fever in under 5 s: assessment and initial management [20]	2019	National Institute for Health and Care Excellence	GRADE
United States of America	Management of fever without source in infants and children [21]	2000	American College of Emergency Physicians	None
	Practice guideline for the management of infants and children 0–36 mon of age with fever without source [22]	1993	American Academy of Pediatrics	Modified Delphi
	Evaluation and management of well-appearing febrile infants 8 to 60 d [23]	2021	American Academy of Pediatrics	AHRQ
Australia	Children and infants with fever [24]	2020	New South Wales Government	NHMRC designation of levels of evidence
	Fever in children aged 1–2 mon [25]	2020	South Australian Pediatric Clinical Practice	None
	Febrile illness: emergency management in children [26]	2019	Children’s health Queensland Hospital and Health Service	None
	Fever without source [27]	2020	Government of Western Australia–Child and Adolescent Health Service	None
Canada	Fever in young infants [28]	2019	Translating Emergency Knowledge for Kids	None

*AGREE II* Appraisal of Guidelines for Research and Evaluation Instrument, *GRADE* Grading of Recommendations Assessment, Development and Evaluation, *AHRQ* Agency for Healthcare Research and Quality, *NHMRC* National Health and Medical Research Council

### Guideline definitions

An overview of the guideline definitions is shown in Table 2. The age range of the population as reported by the guidelines varied widely. The target age population of two guidelines was 0–2 months, compared to 0 months to 16 years according to the Dutch guideline [19, 23, 28]. Fever was defined as a temperature of ≥ 38.0 ℃ by seven of ten guidelines [20–23, 25, 26, 28]. There was no definition of FWS described in seven of ten guidelines [19, 20, 23–26, 28]. The guideline was applicable to both children with a low risk and a high risk of serious infection in nine of ten guidelines [19–22, 24–28]. The guideline of the American Academy of Pediatrics (AAP) was the only guideline applicable exclusively to children with a low risk of serious infection [23]. Recognizing serious infection was an objective in seven of ten guidelines [19, 20, 22, 24–26, 28].

**Table 2:** Guideline definitions

Variables	The Netherlands	UK	USA			Australia				Canada	
	NVK [19]	NICE [20]	ACEP [21]	AAP [22]	AAP [23]	NSW [24]	SA [25]	CHQ [26]	CAHS [27]	TREKK [28]	
Age of population											
0–2 mon					√					√	
1–2 mon							√				
0–3 y			√	√							
0–5 y		√				√					
0–> 3 mon								√	√		
0–16 y	√										
Definition of fever											
≥ 38.0 ℃		√	√	√	√		√	√		√	
> 38.0 ℃	√					√			√		
Definition of fever without source											
Not available	√	√			√	√	√	√		√	
No source of infection is apparent after a thorough examination in a nontoxic infant or child without significant underlying illness			√								
An acute febrile illness in which the etiology of the fever is not apparent after a careful history and physical examination^a^				√					√		
Target population of FWS											
Applicable for low-risk group only					√						
Applicable for high-risk group only											
Applicable for high- and low-risk groups	√	√	√	√		√	√	√	√	√	
Definition of serious infection											
Not available					√				√	√	
Meningitis	√	√	√			√	√	√			
Sepsis	√	√		√			√				
Bacteremia			√				√	√			
Urinary tract infection	√	√	√	√		√	√	√			
Pneumonia	√	√	√	√			√	√			
Enteritis				√			√				
Septic arthritis	√	√		√			√	√			
Osteomyelitis	√	√		√			√	√			
Encephalitis	√	√									
Kawasaki disease	√	√				√		√			
Objectives											
Recognizing serious infection	√	√		√		√	√	√		√	
Minimizing diagnostics	√					√		√		√	
Evidence management of FWS			√		√						
Improving clinical assessment of FWS		√			√					√	
Decreasing variation in care					√						

*FWS* fever without a source, *NVK* Dutch Association of Pediatrics, *NICE* National Institute for Health and Care Excellence, *AAP* American Academy of Pediatrics, *ACEP* American College of Emergency Physicians, *NSW* New South Wales, *SA* South Australian, *CHQ* Children’s Health Queensland Hospital and Health Service, *CAHS* Child and Adolescent Health Service, *TREKK* Translating Emergency Knowledge for Kids. ^a^Definition in broad terms may differ from the precise definition in the guideline

To distinguish recommendations for children with a low, intermediate or high risk of serious infection, the guidelines used multiple clinical criteria (Supplementary Table 2). We found a wide variation in clinical criteria. Most guidelines defined a high risk of serious infection in case of a pale or mottled skin, lethargy or drowsiness, grunting or tachypnea [19, 20, 24–27]. Criteria mentioned as classifying for a low risk are less consistent, but mostly included birth after 37 weeks of gestation and a nontoxic clinical condition. In total, 20 clinical criteria were mentioned defining the low-risk group, 25 defining the intermediate risk group and 36 defining the high-risk group.

### Diagnostic guideline recommendations

Diagnostic recommendations of each guideline are shown in Table 3. There are a number of consistencies between the guidelines. For all children irrespective of the clinical condition younger than one month of age and 1–3 months old, laboratory testing of WBC or CRP was recommended by most guidelines [19, 20, 25–28]. Furthermore, seven of nine guidelines recommended a blood and urine culture in all children younger than one month [20–22, 24, 26–28]. In children older than three months, guidelines recommended performing a blood culture [20–22, 24, 26, 27] and considering CSF analysis exclusively in case of a high risk of serious infection [19, 20, 24, 27].

**Table 3:**
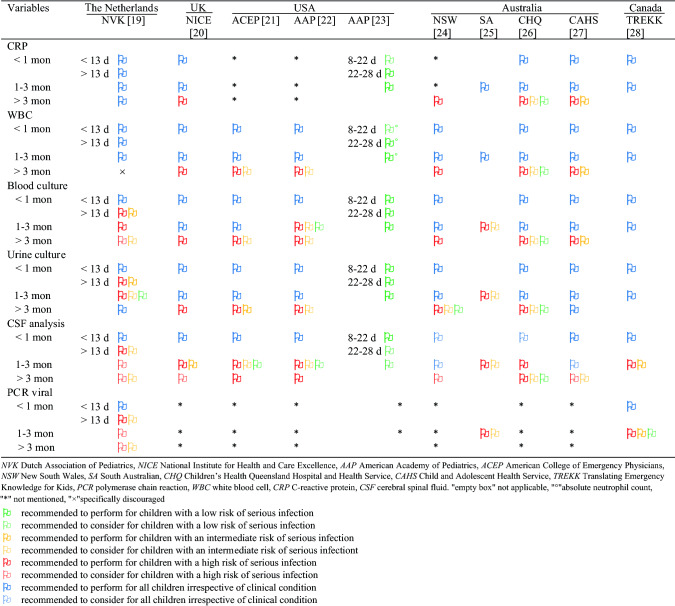
Diagnostic recommendations and considerations

Besides consistencies, we also found a number of differences in diagnostic recommendations. In children between one and three months old, six guidelines [20, 21, 24, 26–28] recommended to perform blood culture irrespective of the clinical condition compared to three guidelines [19, 22, 25] who recommended to perform blood culture exclusively in case of a high risk of serious infection. There was also disagreement in urine cultures in children older than three months with some guidelines only recommending a urine culture for children with a high risk of serious infection [20–22, 24, 26], while other guidelines recommended a urine culture irrespective of the clinical condition [19, 27]. In children between one and three months old, recommendations for CSF analysis varied from considering it in all children or considering in children with high risk of serious infection, to performing in all children with high risk of serious infection. Also, three of ten guidelines recommended to perform PCR for viral pathogens [19, 25, 28], while seven guidelines do not mention any recommendations [20–24, 26, 27]. Finally, instead of CRP measurement, three of ten guidelines described the diagnostic value of incorporating procalcitonin in future guidelines and advise further research [19, 20, 28].

### Therapeutic guideline recommendations

Therapeutic recommendations of each guideline are shown in Table 4. Almost all guidelines recommended antibiotic treatment for children younger than one-month-old, irrespective of the clinical condition [19–22, 24–28]. There was agreement for antibiotic treatment in children older than three months: all guidelines recommended treating or considering antibiotic treatment exclusively in case of high risk of serious infection [19–28]. Differences in antibiotic treatment was seen in children aged 1–3 months old. Seven guidelines recommended antibiotic treatment in children with high risk of serious infection, compared to two guidelines who recommended antibiotic treatment in children irrespective of the clinical condition [19–22, 25, 26, 28]. Four guidelines did not mention acyclovir treatment [21, 22, 26, 27], while six guidelines recommended to consider acyclovir treatment only in children with high risk of serious infection, irrespective of age [19, 20, 23–25, 28].

**Table 4:**
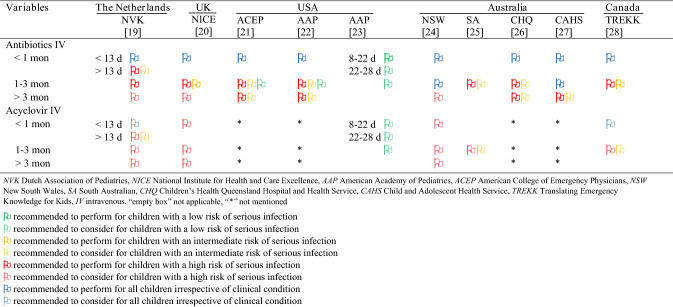
Therapeutic recommendations and considerations

## Discussion

In this study we compared the definitions and diagnostic and treatment recommendations of national and regional FWS guidelines of five high-income countries. We found these guidelines broadly consistent, especially for children younger than one month. The reported age range of children with FWS varied widely. Differences were seen most in recommendations for children aged 1–3 months and above three months of age in performing microbiologic cultures, CSF analysis and in antibiotic treatment. This knowledge may be of assistance to future guideline development.

We found consistency across the included FWS guidelines, particularly in children younger than one month with FWS with most guidelines advising CRP testing and antibiotic treatment irrespective of the clinical condition. This agreement in managing young children is also reflected in clinical practice. Among 37 emergency departments in the USA most consistency was reported in laboratory testing in children younger than one month, compared to substantial variation in children aged 1–2 months and 2–3 months. A similar inverse association between age and practice variation in antibiotic treatment was reported by Aronson et al. [9]. Our study found agreement among all guidelines in antibiotic treatment of children with high risk of serious infection younger than three months of age. Moreover, consistency was seen in a sepsis work-up for children older than three months of age meaning all guidelines recommended to perform or consider a blood culture, CSF analysis and antibiotic treatment exclusively in children with a high risk of infection. These findings implicate that most guidelines adopt a similar careful approach in neonates while advising a higher threshold to extensive diagnostic and therapeutic management in children aged above three months. This approach is understandable, considering the higher risk of bacterial infection in neonates compared to older children [4–6].

The results of this study also show important differences between FWS guidelines, particularly for children older than one month. In children aged above three months there was particular disagreement in when to perform basic diagnostic testing, whereas in children aged 1–3 months guidelines were inconsistent in when to perform a sepsis workup. This is in line with the previously mentioned variation in performed CSF analysis, with rates ranging between hospitals from 40% to 90% of children with FWS aged 1–3 months [14, 29]. This guideline inconsistency and concurrent practice variation reflect the diagnostic dilemma of the age category in between the young neonate with an elevated risk of serious infection, and the older child with a lower risk and a decreasing trend in extensive diagnostic testing [30]. Guidelines may partially differ due to geographic differences in primary and secondary health care systems, antibiotic use and resistance patterns. Weighing risks and benefits of extensive testing and empirical antibiotic treatment may also be influenced by cultural opinions and preferences of physicians and parents [31, 32]. However, we also reported differences between guidelines from the same country. Another reason for differences between guidelines is the lack of international consensus in definitions of FWS, potential serious infections and relevant age ranges. This lack has not been addressed in literature regarding FWS as much as for neonatal sepsis. Similarly for neonatal sepsis, lack of consensus in definitions of FWS hampers ongoing collaborative research and benchmarking for guideline development [33]. For instance, the targeted age range varied widely. Despite multiple studies reporting a drastic step-wise decrease of serious bacterial infection after the first week of life, most guidelines still classified all children younger than one month as high risk [23]. Third, the development and implementation of new diagnostic methods also contributes to differences: the use of PCR to detect viruses for example was only mentioned by a few guidelines. As (respiratory) viruses are a frequent cause of FWS, overuse of antibiotics is likely to decrease when viral testing is addressed in FWS guidelines and should therefore be included [34–36].

Inconsistency between FWS guidelines has important consequences, contributing to increased practice variation. Aronson et al. evaluated the association between guideline inconsistency and practice variation among hospitals in the USA. The FWS recommendations from 21 separate hospital guidelines contained much variance, which correlated with the observed practice variation [9]. Moreover, adherence to FWS guidelines in the Netherlands was only 50% which indicates room for improving implementation of guideline recommendations or the recommendations themselves [13]. An Australian study showed a wide range of adherence across FWS recommendation categories and age groups [37]. They measured lower adherence in older children, where our findings stated most inconsistency between the Australian guidelines in children older than one month. Studies of barriers to guideline adherence reported several factors influencing physicians, including lack of agreement with recommendations, doubts about the scientific grounds or lack of outcome expectancy, complicated description of recommendations and inconsistency between similar guidelines [15, 38]. Our findings corroborate several of these barriers in FWS guidelines, besides the inconsistency between guidelines. The majority did not report an established method of grading scientific evidence supporting their recommendations, which may increase doubts among physicians. While most guidelines were updated in the last two years, the AAP guideline for FWS applicable to children with both a low and high risk for serious infection was published in 1993 [22]. Recently, the AAP published a new guideline applicable for the well-appearing child with FWS, yet an updated guideline applicable for children with a high risk is still lacking [23]. This is likely to contribute to the aforementioned variance between the 21 separate FWS hospital guidelines [9]. Therefore, our findings and these studies indicate several aspects that could improve guideline adherence such as decreasing inconsistency between guidelines, particularly within countries, using established grading methods and regularly updating guidelines.

The findings of the current study can assist in harmonizing guideline development and future research for the management of children with FWS. Despite many publications on risk assessment tools and practice guidelines, the appropriate management of children with FWS still remains a highly debated and studied topic. In guideline development, it is common to perform a search of existing guidelines regarding specific management and compare it with the latest evidence to compose a recommendation. Subsequently, the aim is to provide evidence-based practical guidelines, improving quality of care and reducing unwanted variation. It is not necessary or advisable to aim for complete harmonization between national guidelines, as practical considerations and local applicability are also taken into account. Differences between health care systems or resistance patterns in high-income countries can provide solid arguments for international differences between guidelines. It is, however, very likely that many recommendations are based on the same available evidence. To support interpretation and comparison of evidence for guideline development, it is recommended to establish international consensus on targeted age groups and definition for FWS and potential serious infections. Furthermore, identifying significant differences between guidelines provides insight in FWS recommendations lacking consensus or lacking valid scientific grounds and may reveal important opportunities for further analysis and increasing adherence.

We acknowledge that this study contains several limitations. Although our literature search enabled a comparison of guidelines from various countries, we may have missed potential eligible guidelines due to the exclusion of non-English or Dutch guidelines and possible lack of access to guidelines or guidelines which are not published publically. We did not use translation programs to include guidelines in more languages, since the interpretation of health care recommendations require a detailed understanding of the language and may be prone to mistakes. However, our detailed description of FWS definitions and recommendations can easily be compared to a physician’s own local guidelines. Furthermore, our study does not include a guideline quality assessment. This was a deliberate decision since our aim was not to compare the quality of guidelines, but rather to provide insight in current existing recommendations and reveal important differences between guidelines.

In conclusion, national and regional FWS guidelines of high-income countries for management of children are broadly consistent. However, substantial differences were found in diagnostic and treatment recommendations for children aged 1–3 months and above three months. In the context of considerable variation in current practice and guideline adherence, our results imply a need for consistent, effective and practical recommendations for children with FWS aged older than one month. International consensus in age range, definition and management of FWS could improve future guideline development and research efforts. Further research should be undertaken to investigate what scientific or practical reasoning drives the differences between guidelines and evaluate if consensus between guidelines is needed.

## Supplementary Information

Below is the link to the electronic supplementary material.

Supplementary file 1 (DOCX 301 KB)

## Data Availability

The data that support the findings of this study are openly available in figshare.
