# Supplementary material for: Fever without a source in children: international comparison of guidelines
Source: World J Pediatr. 2022 Oct 26;19(2):120–8. doi: 10.1007/s12519-022-00611-8 (PMC9928815; doi:10.1007/s12519-022-00611-8)
Supplement: Supplementary file 1 — (DOCX 301 KB) [file 12519_2022_611_MOESM1_ESM.docx]

| Concept | Search terms |
| --- | --- |
| Children | “Infant” OR “newborn” OR “neonate” OR “neonates” OR “baby” OR “babies” OR “infant” OR “Children” OR “Child” OR “minors” OR “pediatric” |
| Fever | “Fever” OR “febril” OR “febrile” OR “hyperterm” |
| Guideline | “Guideline” OR “Guidelines” |

**Table 1.** Search terms for PubMed, EBSCOhost and Web of Science database search

The concepts were combined as follows: “children” AND “fever” AND “guideline”


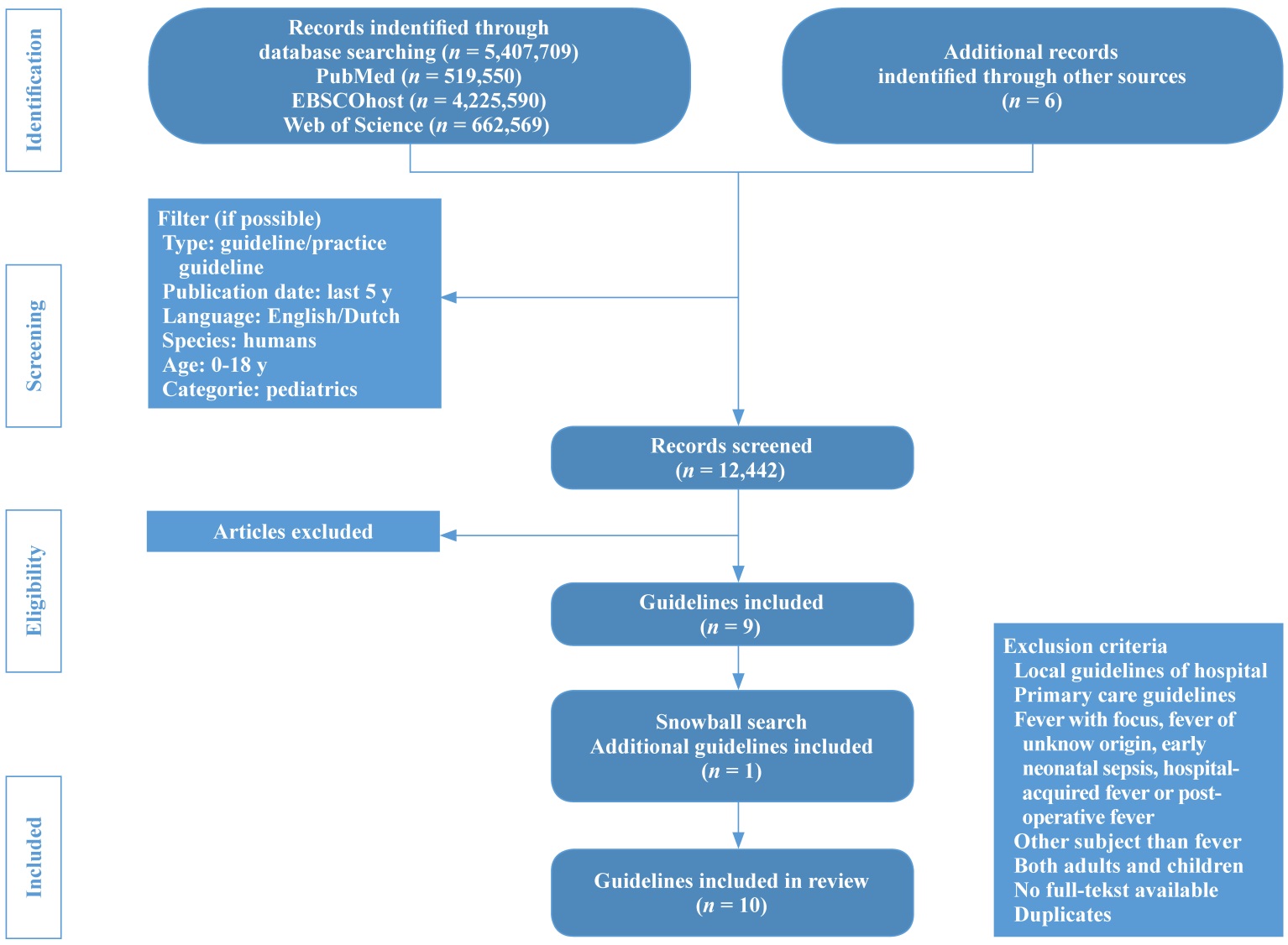


**Fig. 1** Flowchart of the literature search

**Table 2.** Overview clinical criteria for low, intermediate and high risk of serious disease per guideline

| Country | The Netherlands | UK | USA | | | Australia | | | | Canada |
| --- | --- | --- | --- | --- | --- | --- | --- | --- | --- | --- |
| Guideline | NVK [19] | NICE [20] | ACEP [21] | AAP [22] | AAP [23] | NSW [24] | SA [25] | CHQ [26] | CAHS [27] | TREKK [28] |
| Low risk |  |  |  |  |  |  |  |  |  |  |
| Normal color of skin, lips and tongue | X | X |  |  |  |  | X |  | X |  |
| Responds normally to social cues | X | X |  |  |  |  | X |  | X |  |
| Content/smiles | X | X |  |  |  |  | X |  | X |  |
| Stays awake of awakes quickly | X | X |  |  |  |  | X |  | X |  |
| Strong normal cry of not crying | X | X |  |  |  |  | X |  | X |  |
| Normal work of breathing |  |  |  |  |  |  | X |  | X |  |
| Normal skin and eyes | X | X |  |  |  |  | X |  | X |  |
| Moist mucous membranes | X | X |  |  |  |  | X |  | X |  |
| Previous healthy |  |  | X | X |  |  |  |  |  |  |
| Born after 37 wk of gestation | X | X | X |  |  |  | X |  |  | X |
| No prior hospitalization |  |  |  |  |  |  |  |  |  | X |
| No prolonged newborn nursery care |  |  |  |  |  |  |  |  |  | X |
| Uncomplicated newborn nursery care |  |  | X |  |  |  |  |  |  |  |
| Nontoxic clinical appearance | X | X | X | X |  |  |  |  |  |  |
| No intermediate or high risk signs | X | X |  |  |  |  |  |  | X |  |
| No focal bacterial infection on examination (except otitis media) | X | X | X | X |  |  |  |  |  |  |
| No evidence of any infection clinically | X | X |  |  |  |  | X |  |  |  |
| No unexplained jaundice | X | X |  |  |  |  |  |  |  | X |
| No chronic illness | X | X |  |  |  |  | X |  |  | X |
| No prior antibiotics | X | X |  |  |  |  | X |  |  | X |
| Intermediate risk |  |  |  |  |  |  |  |  |  |  |
| Pallor of skin, lips or tongue, or reported by parent or carer | X | X |  |  |  |  | X | X | X |  |
| Not responding normally to social cues | X | X |  |  |  |  | X | X | X |  |
| No smile | X | X |  |  |  |  | X | X | X |  |
| Wakes only with prolonged stimulation | X | X |  |  |  |  |  | X | X |  |
| Decreased activity | X | X |  |  |  |  | X | X |  |  |
| Irritable |  |  |  |  |  |  | X |  |  |  |
| Not strong cry |  |  |  |  |  |  |  | X |  |  |
| Appearing ill to a healthcare professional | X |  |  |  |  |  |  |  |  |  |
| Disease course different from previous diseases | X |  |  |  |  |  |  |  |  |  |
| Nasal flaring | X | X |  |  |  |  |  | X | X |  |
| Tachypnea | X | X |  |  |  |  |  | X | X |  |
| Oxygen saturation ≤ 95% | X | X |  |  |  |  |  | X | X |  |
| Auscultation: crepitations | X | X |  |  |  |  |  | X | X |  |
| Tachycardia | X | X |  |  |  |  |  | X | X |  |
| Dry mucous membranes | X | X |  |  |  |  | X |  | X |  |
| Poor feeding in infant | X | X |  |  |  |  | X | X | X |  |
| Reduced urine output | X | X |  |  |  |  | X | X | X |  |
| Capillary refill time ≥ 3 s | X | X |  |  |  |  |  | X | X |  |
| Rigors | X | X |  |  |  |  |  | X | X |  |
| Fever > 5 d | X | X |  |  |  |  |  |  | X |  |
| Swollen joint | X | X |  |  |  |  |  | X | X |  |
| New lump > 2 cm |  |  |  |  |  |  | X |  |  |  |
| Non-use of a limb | X | X |  |  |  |  |  |  | X |  |
| Unable to bear weight | X | X |  |  |  |  |  |  | X |  |
| Age 3-6 mon, temperature ≥ 39.0 ℃ |  | X |  |  |  |  |  |  | X |  |
| High risk |  |  |  |  |  |  |  |  |  |  |
| Pale/mottled/ashen/blue skin, lips or tongue | X | X |  | X |  | X | X |  | X |  |
| No response to social cues | X | X |  |  |  |  | X |  | X |  |
| Altered mental state |  |  |  |  |  |  |  | X |  |  |
| Lethargy |  |  |  | X |  | X |  | X |  | X |
| Irritability |  |  |  |  |  | X |  | X |  |  |
| Decreased activity |  |  |  |  |  | X |  |  |  |  |
| Decreased alertness |  |  |  |  |  | X |  |  |  |  |
| Appearing ill to a healthcare professional |  | X |  |  |  |  | X |  | X |  |
| Does not wake or if roused does not stay wake | X | X |  |  |  |  | X |  | X |  |
| Weak, high-pitched or continuous cry | X | X |  |  |  | X | X | X | X |  |
| Grunting | X | X |  |  |  |  | X | X | X |  |
| Hypoventilation or hyperventilation |  |  |  | X |  |  |  |  |  |  |
| Tachypnea | X | X |  |  |  | X | X | X | X |  |
| Moderate or severe chest indrawing | X | X |  |  |  | X |  | X | X |  |
| Auscultation: diminished breath sounds |  |  |  |  |  |  |  |  |  |  |
| Signs of poor perfusion |  |  |  | X |  | X |  | X |  |  |
| Cool peripheries |  |  |  |  |  |  | X | X |  |  |
| Bounding pulses or wide pulse pressure |  |  |  |  |  |  | X |  |  |  |
| Reduced skin turgor | X | X |  |  |  |  | X | X | X |  |
| Bilious vomiting |  |  |  |  |  |  | X |  |  |  |
| Decreased fluid intake |  |  |  |  |  | X | X | X |  |  |
| Decreased urine output |  |  |  |  |  | X | X | X |  |  |
| Tachycardia |  |  |  |  |  | X | X | X |  |  |
| Signs of shock |  |  |  |  |  |  |  |  |  | X |
| Neck stiffness | X | X |  |  |  |  |  |  | X |  |
| Bulging fontanelle | X | X |  |  |  |  | X |  | X |  |
| Petechiae | X |  |  |  |  |  | X |  |  |  |
| Non-blanching rash |  | X |  |  |  |  |  |  | X |  |
| Coagulopathy |  |  |  |  |  |  |  |  |  | X |
| Rigors |  |  |  |  |  |  | X |  |  |  |
| Status epilepticus | X | X |  |  |  |  |  |  | X |  |
| Focal seizures | X | X |  |  |  |  | X | X | X |  |
| Focal neurological symptoms | X | X |  |  |  |  |  |  | X |  |
| Age < 1 mon with FWS | X |  |  |  |  |  |  |  |  |  |
| Age < 3 mon with FWS |  | X |  |  |  |  |  |  | X |  |
| Evidence of organ dysfunction |  |  |  |  |  |  |  |  |  | X |

*FWS* fever without a source, *NVK* Dutch Association for Pediatrics, *NICE* National Institute for Health and Care Excellence, *ACEP* American College of Emergency Physicians, *AAP* American Academy of Pediatrics, *NSW* New South Wales, *SA* South Australian, *CHQ* Children's Health Queensland Hospital and Health Service, *CAHS* Child and Adolescent Health Service, *TREKK* Translating Emergency Knowledge for Kids
